# Empowering Latino Communities Through the Colorectal Health Research Champions Model: Enhancing Colorectal Cancer Awareness and Screening Advocacy

**DOI:** 10.1007/s10903-025-01829-0

**Published:** 2025-12-04

**Authors:** Nancy Valencia-Rojas, Katelyn Schifano, Shiva Salehian, Beomchang Kim, Maria D. Thomson, Vanessa B. Sheppard

**Affiliations:** Virginia Commonwealth University, Richmond, USA

**Keywords:** Colorectal cancer awareness, Screening, Disparities, Latinos

## Abstract

Colorectal cancer (CRC) is the second leading cause of cancer mortality among Latinos who remain underrepresented in screening and are more often diagnosed at later stages, leading to poorer outcomes. Barriers include low health literacy, lack of insurance, limited access to screening, and cultural mistrust of healthcare and research. To implement the Colorectal Health Research Champion (CHRC) Model to address CRC screening disparities among immigrant Latinos, primarily from Central and South America, residing in Richmond, VA’s urban Latino community. The CHRC model integrates evidence-based strategies from Screen to Save (S2S), the National Outreach Network Community Health Educator (NON-CHE) program, and Massey Comprehensive Cancer Center’s Community Champion program. Five trained champions conducted peer-to-peer chats on CRC symptoms, risk factors, screening guidelines, healthy behaviors, and clinical trials. Pre and post-surveys were collected from 52 Latino participants. The Wilcoxon signed-rank test assessed changes in knowledge and screening intent. Baseline CRC awareness and screening rates were low. Post-intervention, participants showed notable improvements in recognizing CRC risk factors such as physical inactivity, family history, and the importance of early detection. Participants also demonstrated a better understanding of screening options like the FIT test and expressed willingness to adopt healthier behaviors and pursue screening. However, mistrust and limited understanding of research remained challenges. The CHRC model successfully enhanced CRC knowledge, screening, and early detection awareness among Latinos, while encouraging consideration of clinical trial participation. Success was driven by trusted community champions leveraging social networks, culturally tailored education, addressing research hesitancy, and using flexible outreach strategies.

## Introduction

Colorectal cancer (CRC) is the second leading cause of cancer death in the United States [1] and is also among the leading causes of cancer mortality among Latinos, along with lung and pancreatic cancer [2]. Latinos, who represent 19% of the U.S. population [3] and 10.5% of the population in Virginia [4] have a CRC incidence of 38.2 and 27.5 per 100,000 for men and women, respectively [5]. Unlike long-established Latino enclaves in California, Florida, and Texas, Virginia’s Latino community is relatively new, composed largely of first-generation, Spanish-speaking immigrants from Central and South America who face limited access to insurance and preventive care.

Despite CRC being among the most preventable cancers, Latinos who are 50 years or older are less likely to be screened for CRC, with the screening rates ranging from 48% to 50%, compared to a significantly higher rate of 65% among Whites [2]. Additionally, Latinos are more likely than Whites to be diagnosed at advanced stages of CRC [2], which results in lower life expectancy and a reduced survival rate within five years of diagnosis [6, 7].

Studies have shown that Latinos are less aware of CRC risk factors and prevention strategies, and are more likely to engage in risky behaviors, such as lower screening rates, diets high in processed foods and low in fiber, physical inactivity, and higher obesity rates [8]. Obesity and type 2 diabetes, both linked to higher CRC risk, are more common among Latinos and often worsened by poor diet, low physical activity, and lifestyle changes associated with acculturation upon moving to the U.S [9, 10]. Although healthy habits like exercise, a balanced diet, and avoiding alcohol and smoking can lower the risk [11, 12] many Latinos continue to have high levels of these risk factors. Latinos also consume more alcohol than other immigrant minorities, increasing their CRC risk [2]. Limited access to healthcare and preventive services also contributes to poorer outcomes.

Screenings significantly reduce CRC incidence and mortality by detecting it at its early stages [2]. The Healthy People 2030 goal is to reach a 68.3% screening rate among aged 50–75 [13]; However, only 42% of Latino men and 47.5% of Latino women are up to date far below the 60% rate of White adults [2]. Increasing awareness about CRC screening is essential, as low health literacy remains a major barrier to CRC screening compliance among Latinos [14]. Other factors include language barriers, limited understanding of the healthcare system, low cultural competence among providers, low income, and lack of insurance [15, 16].

The Colorectal Health Research Champions (CHRC) model, developed by Virginia Commonwealth University (VCU) Massey Comprehensive Cancer Center (MCCC), aims to address CRC screening disparities in Central Virginia. It combines three evidence-based educational interventions to improve CRC awareness and promote screening. The first is the Screen-to-Save (S2S), a national initiative to increase CRC screening rates in diverse and rural populations, developed by the Center to Reduce Cancer Health Disparities (CRCHD) at the National Cancer Institute (NCI) [17]. The second is the National Outreach Network (NON) Community Health Educator (CHE) program, which partners with NCI-designated Cancer Centers [18]. The third is MCCC’s Community Champion Program [19], which engages community members to advocate for screening and participation in cancer research trials.

Grounded in social network theory, the CHRC model trains champions to raise awareness of CRC symptoms, risk factors, screening guidelines, healthy behaviors, and the ethical principles of clinical trials. This paper describes the model’s implementation among urban Latinos and shares key lessons, challenges, and opportunities to inform future community-based efforts to reduce CRC disparities.

## Methods

The CHRC model combines three evidence-based interventions: (1) S2S, (2) NON-CHE, and (3) MCCC’s Community Champions Program. S2S is an NCI Colorectal Cancer Outreach and Screening Initiative. The NON-CHE engages community health educators who leverage trust and cultural connections to disseminate culturally tailored, evidence-based cancer information to underserved communities [20]. The S2S/NON-CHE was previously implemented by MCCC, an NCI-designated Cancer Center, in rural and underserved Virginia communities to address disparities in CRC detection, increase awareness, and promote adherence to U.S. Preventive Services Task Force (USPSTF) screening recommendations. The S2S includes an 18-item questionnaire administered before and after educational sessions to assess changes in knowledge, intentions to discuss CRC, and intentions to engage in screening and healthier lifestyle behaviors. It also collects demographic data, screening history, and family history of CRC [20]. Full details of the S2S and S2S/NON-CHE are available elsewhere [20, 21].

**Fig. 1 CHRC Training Content and Agenda:**
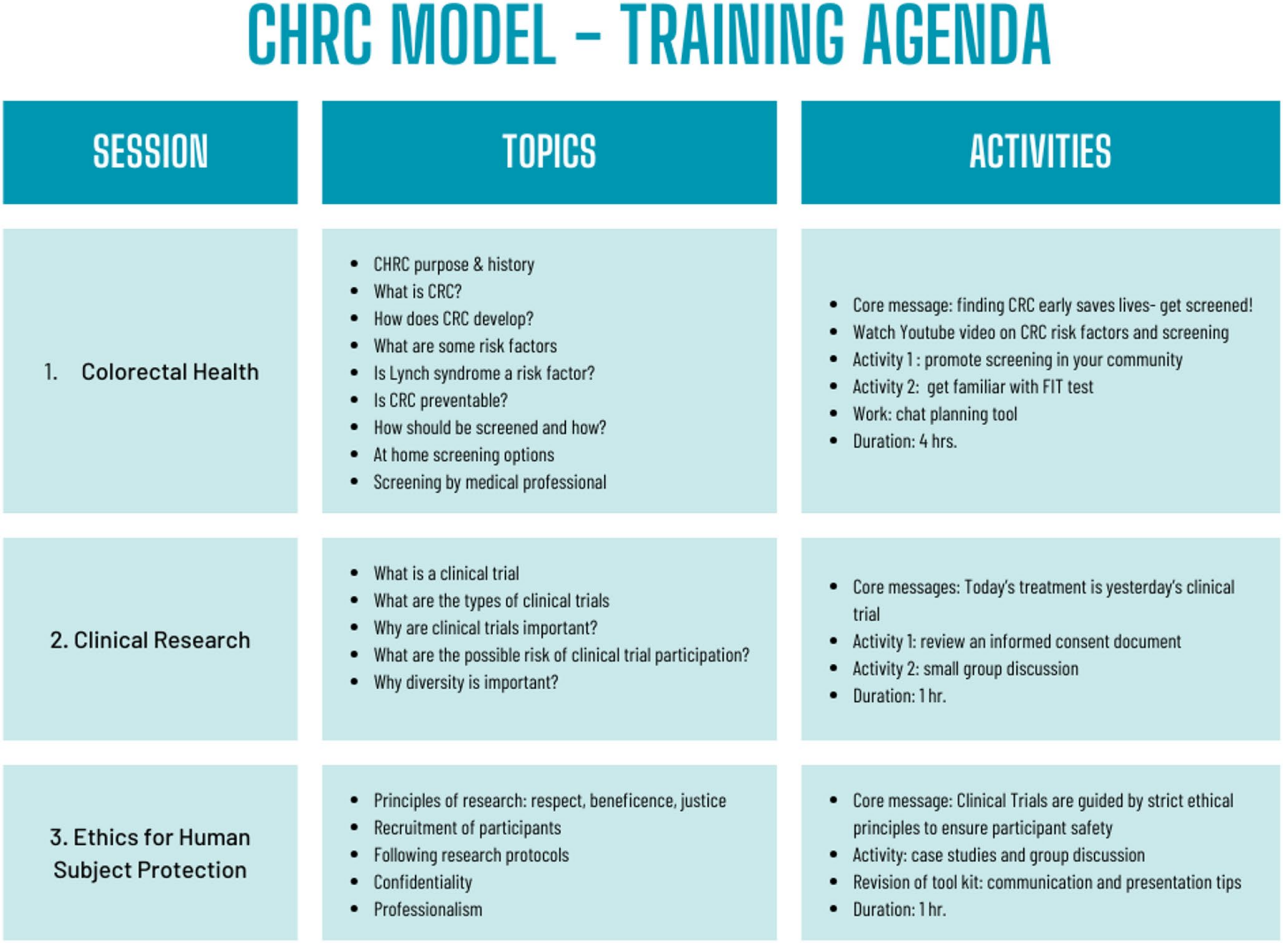
CHRC Training Content and Agenda. Descriptive caption: CHRC Model Training Content and Agenda. Outlines the training structure for the Colorectal Health Research Champion (CHRC) Model. The agenda is divided into three sessions: [1] Colorectal Health [2], Clinical Research, and [3] Ethics for Human Subject Protection. Each session lists key topics and corresponding activities. Topics include CRC purpose, risk factors, screening methods, clinical trials, diversity in research, and ethical principles like respect, confidentiality, and professionalism. Activities combine educational messages, videos, interactive discussions, and tool reviews to prepare champions for community outreach. The training totals six hours: four hours on colorectal health and one hour each on clinical research and ethics

**Fig. 2 Massey Comprehensive Cancer Center's CHRC Implementation Approach:**
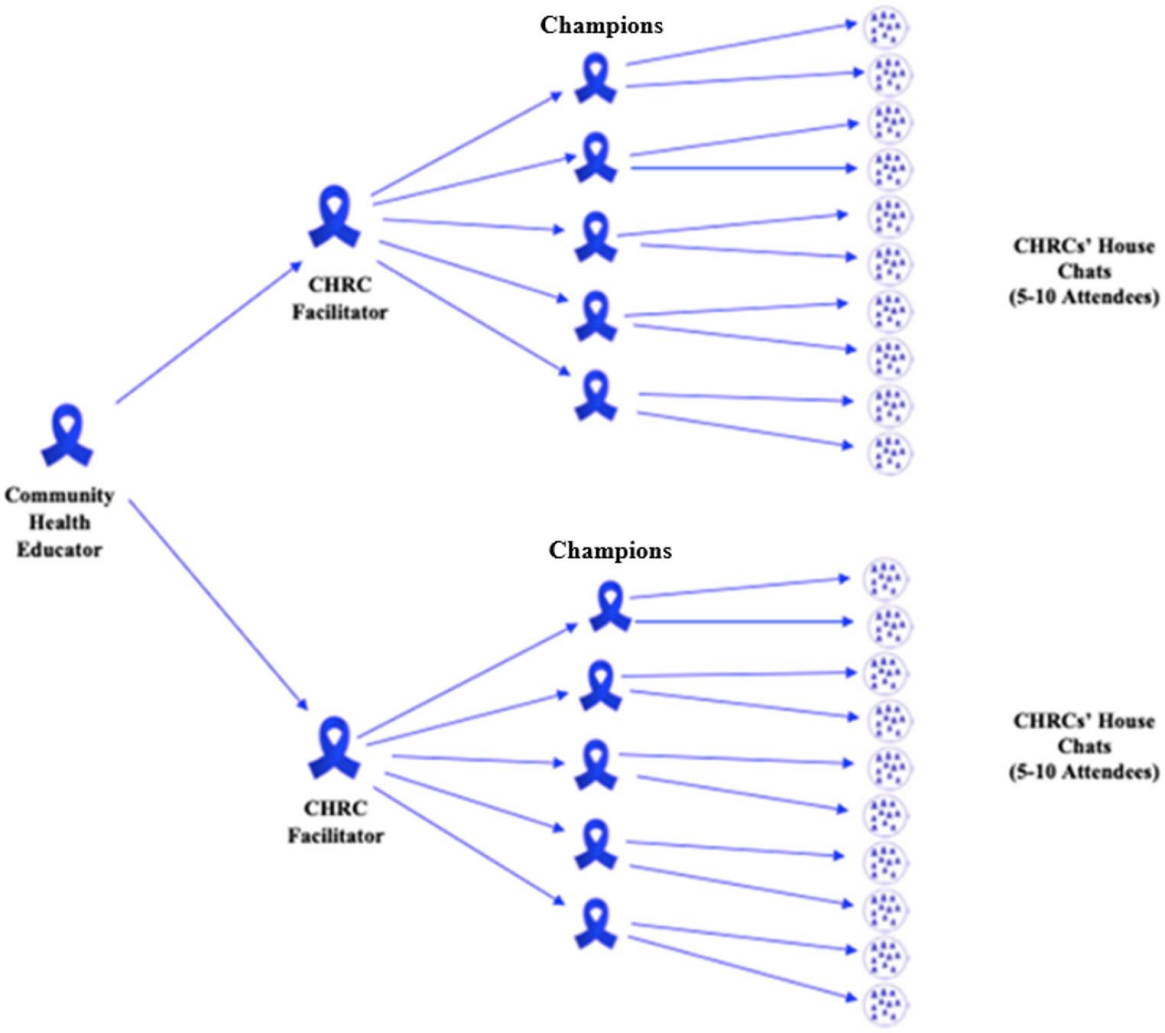
Massey Comprehensive Cancer Center’s CHRC Implementation approach. Descriptive caption: Diagram illustrating the Colorectal Health Research Champion (CHRC) Model’s implementation process. A Community Health Educator trains a CHRC Facilitator, who then train multiple Champions. Each Champion conducts CHRC educational house chats with small groups of 5–10 community members. The visual shows two tiers of Champions delivering peer-to-peer education through their personal networks, emphasizing the model’s grassroots, community-led approach to raising colorectal cancer awareness

MCCC combined the S2S/NON-CHE with its Community Champion Program [19] to create the CHRC model, which trains community members to raise awareness about CRC screening and advocate for clinical trial participation within their social networks. It covers screening guidelines, educates about risks and testing options, connects individuals to local cancer resources, and promotes clinical trial participation. Key adaptations to the original CHRC model for implementation among Latinos are summarized in Table 1. The CHRC training consisted of six hours of instruction, including S2S pre- and post-questionnaires to assess champions’ knowledge, guidance on peer outreach, and feedback to build confidence in delivering content. All five CHWs were trained together, with Sessions 1 and 2 (colorectal health and clinical research) delivered in person on a Saturday afternoon at a public library (5 h), and Session 3 (research ethics) delivered virtually on a Wednesday evening. Detailed content for each session is outlined in Fig. 1. After training, champions were asked to reach out to 10 peers within their social networks through house-chats (i.e., friends, family, neighbors, coworkers). The goal was for champions to complete their outreach within one month. Figure 2. Illustrates the CHRC implementation approach.

*Implementation of CHRC Model*. Five community health workers (CHWs), two men and three women, were trained as champions. A native Spanish-speaking community engagement and research coordinator from VCU delivered the six-hour training held in person and via Zoom, using fidelity checklists to ensure all topics were covered. The CHWs, originally from Bolivia, Nicaragua, and Venezuela, each recruited at least ten peers from their social networks to discuss CHRC educational materials. These networks mostly consisted of family members and friends, though specific data on their composition were not collected. They shared the content through house chats and one-on-one conversations, with each session lasting about an hour. All materials were translated into Spanish and adapted for low literacy to ensure cultural responsiveness. Most conversations occurred in participants’ homes or trusted community spaces, with champions choosing the format that worked best for their networks, typically one-on-one but sometimes in small informal groups.

Pre- and post-S2S questionnaires were administered to collect demographics, CRC knowledge, and intentions to get screened, engage in CRC conversations with their social networks, and adopt healthier behaviors. Demography data included age, ethnicity, education, and insurance status. While the country of origin was not asked, champions confirmed that all participants were native Spanish speakers from Central and South America. Eligibility included self-identifying as a Latino, being 18 years or older, and residing in Richmond, VA. Champions received $300 to cover their time and to purchase snacks or tokens for the participants. Participants received no direct compensation. The study was approved by the VCU Institutional Review Board.

To compare the pre-and post-intervention data, we used the Wilcoxon signed-rank test for paired samples. For the knowledge scores, participants’ unanswered questions were counted as incorrect responses. Intention to engage with social networks and adopt healthier behaviors was measured using a 5-point Likert scale, ranging from “strongly agree – 5” to “strongly disagree – 1” [22] The survey assessed participants’ intentions to get screened for colorectal cancer, discuss CRC with family or friends, and the importance of minority group participation in clinical trials. All statistical analyses were conducted using R version 4.4.2, with a significance level of 0.05 [23] To determine if the intervention positively impacted CRC knowledge, clinical trial knowledge, and behavioral intentions, we used a one-sided test (right-tailed test) (Table 1).

## Results

Five champions reached 52 Latino individuals over three weeks. Participants ranged in age from 20 to 70 years, with 20 (38%) aged 45 or older. 60% were female, and 65% had a high school diploma or less. All were born outside the U.S. and spoke Spanish with limited English proficiency. Most participants had limited access to health insurance or a primary care provider. Among those eligible for CRC screening, only four (7.7%) had previously been screened. Table 2 summarizes participants demographics.

**Table 1:** Key Adaptations to the original Colorectal health research champion Model

	Original S2S	Adaptation	Rationale
Data collection	Online	Pen and paper	Latinos with low educational level prefer to read long questionnaires in pen and paper format.
Training	4 sessions (in-person)	4 sessions (3 in-person, 1 online)	To facilitate attendance (In-person sessions were held on weekends, while the online session took place on a weekday evening)
Recruitment	Flyers, email blasts, with community partners,	Through champions’ social networks and community partner	Latinos have a high level of trust in community health workers.
Administration	Requires SSN^1^, home address, phone number	Identifiers were not required	To facilitate recruitment

^1^Social Security Number

**Table 2:** Participant demographic characteristics (*N* = 52)

	*n* (%)
Age Mean (Min-Max; SD)	42(22–70; 12.2) NA:1
Gender	
Female Male	31 (59.6%)41 (40.4%)
Education	
8 grade or less Some high school High school graduate Some college College degree Unknown	10 (19.2%) 12 (23.1%) 12 (23.1%) 8 (15.4%) 9 (17.3%) 1 (1.9%)
Insurance status	
Uninsured Through work Individual/Private Medicare Medicaid Unknown	34 (65.4%) 3 (5.8%) 5 (9.6%) 1 (1.9%) 4 (7.7%) 5 (9.6%)

We summarized participant survey responses before and after the intervention for CRC knowledge (Table 3); social engagement and willingness to adopt healthy behaviors (Table 4); knowledge about clinical trials (Table 5); and intentions to participate in clinical trials (Table 6). In this context, “social engagement” refers to participants’ willingness to discuss CRC risk factors and screening options with family and friends.

**Table 3:** Differences in knowledge about colorectal cancer (*N* = 52)

Variables	Pre-test *n* (%)	Post-test *n* (%)
The organ where CRC^1^ starts		
Colon & rectum Liver Stomach Small intestine Not Available	24 (46.2%) 2 (3.8%) 12 (23.1%) 15 (28.8%) 1 (1.9%)	47 (90.4%) 0 (0%) 0 (0%) 5 (9.6%) 0 (0%)
What does FIT^2^ detect		
Blood Fat Tumors Polyps Not Available	16 (30.8%) 21 (40.4%) 9 (17.3%) 12 (23.1%) 3 (5.8%)	31 (59.6%) 6 (11.5%) 1 (1.9%) 18 (34.6%) 1 (1.9%)
Risk factors for CRC		
Poor diet Smoking Lack of physical activity Heavy drinking Overweight Not Available	41 (78.8%) 18 (34.6%) 8 (15.4%) 12 (23.1%) 15 (28.8%) 1 (1.9%)	50 (96.2%) 48 (92.3%) 46 (88.5%) 43 (82.7%) 41 (78.8%) 5 (9.6%)
Eligible age for colonoscopy screening		
25 35 45 55 Not Available	6 (11.5%) 12 (23.1%) 16 (30.8%) 14 (26.9%) 4 (7.7%)	2 (3.8%) 1 (1.9%) 45 (88.5%) 1 (1.9%) 2 (3.8%)
Eligible age for FIT test		
25 35 45 55 Not Available	15 (28.8%) 17 (32.7%) 13 (25.0%) 6 (11.5%) 1 (1.9%)	14 (26.9%) 11 (21.2%) 26 (50.0%) 1 (1.9%) 0 (0.0%)
Family history of Lynch Syndrome raises CRC risk		
True False Not Available	16 (30.8%) 28 (53%) 8(15.4%)	46 (88.5%) 5 (9.6%) 1 (1.9%)
Early detection leads to higher survival		
True False Not Available	45 (86.5%)7 (13.5%)0 (0.0%)	51 (98.1%)0 (0.0%)1(0.2%)
CRC may be present without symptoms		
True False Not Available	28 (53.8%) 20 (38.5%) 4 (7.7%)	48 (92.3%) 1 (1.9%) 3 (5.8%)
Polyps are precursors of CRC		
True False Not Available	36 (69.2%)9 (17.3%)7 (13.5%)	48 (92.3%)1 (1.9%)3 (5.8%)
Colonoscopy can detect polyps		
True False Not Available	35 (67.3%) 14 (26.9%) 3 (5.8%)	51 (98.1%) 0 (0.0%) 1 (1.9%)
Processed meats are high risk for CRC		
True False Not Available	35 (67.3%) 3 (25.0%) 4 (7.7%)	47 (90.4%) 4 (7.7%) 1 (1.9%)
It’s okay to skip CRC screening if there are no symptoms		
True False Not Available	29 (55.8%)21 (40.4%)2 (3.8%)	7 (13.5%)44 (84.6%)1 (1.9%)
Family history of CRC is a risk factor		
True False Not Available	29 (55.8%) 20 (38.5%) 3 (5.8%)	49 (94.2%) 2 (3.8%) 1 (1.9%)
Physical activity is not a risk factor for CRC		
True False Not Available	18 (34.6%) 30 (57.7%) 4 (7.7%)	16 (30.8%) 32 (61.5%) 4 (7.7%)

^1^Colorectal Cancer; ^2^Fecal immunochemical test (FIT)

**Table 4:** Differences in social engagement and willingness to adopt healthier behaviors (*N* = 52)

Variables	Pre-test *n* (%)	Post-test *n* (%)
Likely to talk with healthcare providers about screening		
Strongly Agree Agree Unsure Disagree Strongly Disagree No response	11(21.2%) 13 (25.0%) 16 (30.8%) 9 (17.3%) 2 (3.8%) 1 (1.9%)	19 (36.5%) 19 (36.5%) 6 (11.5%) 5 (9.6%) 0 (0%) 3 (5.8%)
Likely to get screened		
Strongly Agree Agree Unsure Disagree Strongly Disagree No response	10 (19.2%)17 (32.7%)12 (23.1%)8 (15.4%)1 (1.9%)4 (7.7%)	16 (30.8%)25 (48.1%)6 (11.5%)2 (3.8%)0 (0%)3 (5.8%)
Likely to talk with family, friends		
Strongly Agree Agree Unsure Disagree Strongly Disagree No response	10 (19.2%) 18 (34.6%) 11 (21.2%) 8 (15.4%) 1 (1.9%) 4 (7.7%)	17 (32.7%) 23 (44.2%) 5 (9.6%) 2 (3.8%) 0 (0%) 5 (9.6%)
Likely to eat healthier		
Strongly Agree Agree Unsure Disagree Strongly Disagree No response	16 (30.8%) 22 (42.3%) 8 (15.4%) 3 (5.8%) 1 (1.9%) 2 (3.8%)	24 (46.2%) 21 (40.4%) 2 (3.8%) 1 (1.9%) 0 (0%) 4 (7.7%)
Likely to increase physical activity		
Strongly Agree Agree Unsure Disagree Strongly Disagree No response	13 (25.0%) 26 (50.0%) 6 (11.5%) 3 (5.8%) 2 (3.8%) 2 (3.8%)	25 (48.1%) 21 (40.4%) 2 (3.8%) 0 (0%) 0 (0%) 4 (7.7%)

**Table 5:** Participants’ knowledge of clinical trials (*N* = 52)

Variables	Pre-test *n* (%)	Post-test *n* (%)
CT^1^ tests new treatment for safety		
True False Not Available	31 (59.6%) 15 (28.8%) 6 (11.5%)	42 (80.8%) 4 (7.7%) 6 (11.5%)
Patient always receives the new treatment in a CT		
True False Not Available	23 (44.2%) 25 (48.1%) 4 (7.7%)	42 (80.8%) 5 (9.6%) 5 (9.6%)
Consent form explains CT risks		
True False Not Available	31 (59.6%) 16 (30.8%) 5 (9.6%)	41 (78.8%) 5 (9.6%) 6 (11.5%)
Must stay in CT until the doctor says otherwise		
True False Not Available	28 (53.8%) 20 (38.5%) 4 (7.7%)	18 (34.6%) 27 (51.9%) 7 (13.5%)
Experts and community review CT safety		
True False Not Available	44 (84.6%) 1 (1.9%) 7 (13.5%)	44 (84.6%) 1 (1.9%) 7 (13.5%)
CT assesses if treatment works		
True False Not Available	44 (84.6%) 4 (7.7%) 4 (7.7%)	47 (90.4%) 0 (0%) 5 (9.6%)
Treatment works the same for everyone		
True False Not Available	16 (30.8%) 30 (57.7%) 6 (11.5%)	6 (11.5%) 39 (75.0%) 7 (13.5%)
CT determine optimal medication doses		
True False Not Available	42 (80.8%) 6 (11.5%) 4 (7.7%)	44 (84.6%) 1 (1.9%) 7 (13.5%)
Joining CT is possible only if other options aren’t feasible		
True False Not Available	27 (51.9%) 19 (36.5%) 6 (11.5%)	22 (42.3%) 23 (44.2%) 7 (13.5%)
Potential benefit: access to new or better treatments		
True False Not Available	37 (71.2%) 9 (17.3%) 6 (11.5%)	45 (86.5%) 1 (1.9%) 6 (11.5%)
All side effects are known before trial starts		
True False Not Available	18 (34.6%) 28 (53.8%) 6 (11.5%)	22 (42.3%) 24 (46.2%) 6 (11.5%)

^1^Clinical trials

**Table 6:** Differences in intention to participate in clinical Trials - CT (*N* = 52)

Variables	Pre-test *n* (%)	Post-test *n* (%)
Likely to seek information about CT		
Strongly Agree Agree Unsure Disagree Strongly Disagree No response	3 (5.8%) 15 (28.8%) 17 (32.7%) 11 (21.2%) 1(1.9%) 5 (9.6%)	11 (21.2%) 20 (38.5%) 14 (26.9%) 4 (7.7%) 0 (0%) 3 (5.8%)
Likely to search for a CT		
Strongly AgreeAgreeUnsureDisagreeStrongly DisagreeNo response	3 (5.8%)12 (23.1%)17 (32.7%)11 (21.2%)4 (7.7%)5 (9.6%)	7 (13.5%)20 (38.5%)15 (28.8%)6 (11.5%)1 (1.9%)3 (5.8%)
Importance of minority groups participate in CT		
Strongly Agree Agree Unsure Disagree Strongly Disagree No response	4 (7.7%) 18 (34.6%) 17 (32.7%) 5 (9.6%) 3 (5.8%) 5 (9.6%)	12 (23.1%) 21 (40.4%) 12 (23.1%) 3 (5.8%) 1 (1.9%) 3 (5.8%)
Talk to a health care provider about CT		
Strongly Agree Agree Unsure Disagree Strongly Disagree No response	3 (5.8%) 15 (28.8%) 16 (30.8%) 9 (17.3%) 4 (7.7%) 5 (9.6%)	10 (19.2%) 20 (38.5%) 11 (21.2%) 6 (11.5%) 1 (1.9%) 4 (7.7%)
Likely to join a CT		
Strongly AgreeAgreeUnsureDisagreeStrongly DisagreeNo response	5 (9.6%)9 (17.3%)9 (17.3%)14 (26.9%)9 (17.3%)6 (11.5%)	8 (15.4%)14 (26.9%)8 (15.4%)15 (28.8%)2 (3.8%)5 (9.6%)
Likely to talk to family/friends about CT		
Strongly Agree Agree Unsure Disagree Strongly Disagree No response	2 (3.8%) 19 (36.5%) 11 (21.2%) 10 (19.2%) 4 (7.7%) 6 (11.5%)	11 (21.2%) 14 (26.9%) 11 (21.2%) 10 (19.2%) 1 (1.9%) 5 (9.6%)

At baseline, participants had low CRC awareness and screening intentions. Post-intervention, significant improvements were observed. Recognition of early detection benefits increased from 86.5% to 98.1%, physical inactivity as a risk factor from 42.3% to 61.5%, and family history from 55.8% to 94%.

The median pre-intervention CRC knowledge test score for 52 participants was 8 (interquartile range [IQR]: 5), while the median post-intervention score was 18 (IQR: 2). The interquartile range represents the middle 50% of scores, indicating the variability among participants. The results of the Wilcoxon signed-rank test for paired samples showed a significant improvement in CRC knowledge after the CHRC intervention, with post-test scores significantly higher than pre-test scores (V = 47.5, *p* < 0.0001). This indicates that the intervention effectively enhanced participants’ understanding of CRC.

In the pre-test, 53.8% of participants were willing to share CRC information with family and friends, a number that increased to 76.9% in the post-test. 78.9% of participants agreed or strongly agreed to get screened in the post-test, compared to 51.1% in the pre-test. The results of the Wilcoxon signed-rank test for paired samples showed a significant positive effect of the CHRC intervention on both intentions, with participants showing increased likelihood to get screened (W = 777, *p* = 0.0102) and to talk about CRC with family or friends (W = 670.5, *p* = 0.0043).

## Discussion

This study describes the implementation of the CHRC model among the urban Latinos in Richmond, VA, aiming to increase awareness of CRC symptoms, risk factors, screening guidelines, and the ethical principles of clinical trials. Most participants were uninsured with low socioeconomic status and limited CRC knowledge. Post-intervention scores showed improved CRC knowledge and a greater willingness to get screened, talk with peers and adopt healthier behaviors.

Despite efforts, populations such as immigrants and those without insurance continue to experience suboptimal access to preventive care. The 7.7% CRC screening rate in this group is notably lower than other reported estimates, which were already lowest among adults aged 50–54 years (48%), individuals with less than a high school education (52%), the uninsured (30%); and recent immigrants of less than 10 years (26%) [2]. Although preventive colonoscopies are covered under the Affordable Care Act, Medicare, and Medicaid, most of our participants were ineligible due to a lack of insurance. Achieving Healthy People 2030’s 68.3% [13] among Latinos will require reducing access barriers.

Educational gaps and misconceptions are major contributors to screening disparities [24]. Before the intervention, participants had limited awareness of risk factors beyond diet. After participating in the champions’ house chats, most recognized factors such as physical inactivity, smoking, heavy drinking, being overweight, and limited access to screening services significantly increase CRC risk. They also became more aware of available screening options, demonstrating the value of this peer-led approach.

Our findings align with prior S2S research showing that culturally appropriate CRC education improves knowledge, intent to screen, and to adopt healthier behaviors [25, 26]. However, knowledge alone may not increase screening uptake, particularly in underserved groups [27]. Knowledge acquisition may reflect both positive (e.g., health benefits) as well as negative (e.g., cost or complications) aspects of the expected new behavior [27, 28], which can influence decision-making and screening outcomes.

Multiple barriers, including limited English proficiency [29], low health literacy, low acculturation, fear, cost [30] and stigma or misperceptions about colonoscopy [16] further hinder screening uptake. Structural barriers, such as lack of insurance, limited screening services, and low cultural concordance with providers [33], further compound these challenges. Strong patient-provider relationships are key for adherence [25, 26, 28, 33] as primary care providers play a central role in assessing eligibility, recommending tests, and coordinating referrals and follow-up care [26].

Our findings suggest that with the right infrastructure, NCI-designated cancer centers can effectively engage minority populations in their catchment areas through culturally tailored strategies. This underscores the importance of sustained investment in community outreach and engagement (COE), including teams of bilingual community health educators, adequate compensation for trained staff, and ongoing outreach activities tailored to at-risk populations. Building and maintaining such capacity not only ensures sustainability but also aligns with NCI’s expectation that all cancer centers address cancer disparities within their catchment areas.

Raising CRC awareness is only a first step, but it must be paired with expanded access to free or low-cost screening options for uninsured individuals. Latinos are more likely to receive stool-based tests and are less often referred for follow-up colonoscopy after positive results [31]. This gap persists even among Medicaid beneficiaries and free clinic patients, for whom FIT/FOBT is a first-line screening, underscoring the need to address these barriers to improve early detection and survival outcomes in the Latino population [32].

This project prioritized education and empowerment over screening outcomes. While screening evaluation is important, the CHRC model focused on building knowledge and shifting attitudes to lay the foundation for future action. It is noteworthy that several modifications were adopted to make the CHRC model culturally appropriate to Latinos.

## Opportunities

- *Leveraging trusted social networks*

Partnering with the Virginia Community Health Worker Association (VACHWA) helped recruit native Spanish-speaking CHWs whose strong community ties accelerated implementation and facilitated their buy-in. As trusted community members, they effectively leveraged their social networks to recruit participants quickly, completing the activities within a few weeks.

- *Providing culturally appropriate materials*

Tailoring the materials in Spanish for low literacy was critical, as it facilitated comprehension and encouraged open dialogue about sensitive topics. While the S2S offers Spanish resources, additional CHRC topics, such as research ethics, were also translated. Champions were encouraged to personalize their talks, foster a welcoming environment, and address taboos and misinformation openly, which helped increase participant engagement.

- *Reducing administrative barriers*

VCU’s requirement to collect personal information, such as social security numbers, home addresses, and phone numbers, raised concerns. A waiver was requested to avoid deterring participation and build trust, as immigrant Latinos are often reluctant to share such details [33].

- *Adapting to participants’ schedules*

Offering flexible outreach opportunities on evenings and weekends was essential to maximize participation, demonstrating the importance of aligning program delivery with community work and family responsibilities.

## Challenges and Limitations

- *Limited access to FIT tests and services*

Most participants were interested in CRC screening if barriers were removed, highlighting an opportunity to improve uptake. The CHRC model focused solely on education and empowerment and did not provide mechanisms for direct screening access. Those with insurance were directed to seek their primary care providers, while uninsured participants were referred to free clinics. Future implementations should facilitate access to free FIT tests.

- *Sustainability and scalability of the CHRC model*

The implementation of the CHRC model was grant-funded with a predefined sample size requiring MCCC to include at least 40 Spanish-speaking individuals. Ensuring long-term sustainability of the model will require continued involvement of trained champions and integration into existing prevention programs.

- *Community mistrust and research hesitancy*

Some participants were hesitant to join after learning it was a research study, reflecting common barriers in Latino communities, including limited understanding of community-based research and mistrust. Fears about disclosing personal information or providing biological samples were also reported. Champions addressed these concerns by drawing on their training in clinical trial principles to clarify the study’s purpose and ease apprehensions. Although willingness to join trials increased post-intervention (from 26.9% to 42.3%), the modest shift underscores the need for continued efforts to build trust and awareness.

## Lessons Learned

- *Commitment and trust are key*

CHRC implementation was completed in three weeks. Champions showed strong commitment, conducting multiple one-on-one conversations and leveraging connections at a local food bank. Participants were more receptive when approached by trusted community members at “safe” organizations, underscoring the role of trust in research engagement.

- *Leverage cultural values*

Latino-targeted interventions should leverage cultural values like strong social ties, which can drive behavior change [32]. However, social networks alone cannot overcome the economic, cultural, and structural barriers previously mentioned, such as service availability and attitudes toward preventive care [28]. Champions used their networks to accelerate implementation and initiate CRC conversations; this type of social engagement can increase screening participation at both individual and community levels [24, 31], as research shows strong social ties increase screening participation among minority populations [33].

### Limitations of the Analysis

This study was limited to South Richmond, making the findings less generalizable to other Latino groups. Most participants were uninsured, and only 38% were aged 45 or older, limiting applicability to the broader 45–75 screening age group. The questionnaires also lacked information on country of origin, employment, income, marital status, and years in the U.S., all of which could affect screening behaviors. Future studies should explore these factors, assess post-intervention information sharing, and examine the link between intentions and actual screening. Including a control group would strengthen the analysis.

Future research should also incorporate the perspectives of the community champions, as their frontline experiences provide valuable insights into both the strengths and challenges of peer-to-peer conversations. Champions can highlight the aspects of the training that were most useful, the barriers they encountered during outreach, and what additional tools or resources would have helped them engage participants more effectively. Gathering their feedback will be essential for refining educational materials, strengthening the CHRC model, and ensuring that future interventions are even better tailored to the needs of Latino communities.

## Conclusion

The CHRC model merges S2S, NON-CHE, and MCCC’s champion program and effectively engages Latino CHWs in CRC education, reaching 52 participants in just three weeks through trusted social networks. Key factors included leveraging social ties, using culturally appropriate materials, addressing community mistrust and research hesitancy, and adopting flexible administrative processes, with champions serving a role pivotal to the model’s implementation success. Post-intervention results showed significant gains in CRC knowledge and willingness to adopt healthier behaviors, including screening, confirming the CHRC model’s effectiveness in enhancing understanding of CRC-related topics. As the Latino population grows, ensuring their representation in cancer research, clinical trials, and improving access to preventive services remains essential.

## Data Availability

The data that support the findings of this study are stored securely in REDCap at VCU and are available from the corresponding author upon reasonable request.

## References

[1] American Cancer Society. In: Key Statistics for Colorectal Cancer. https://www.cancer.org/cancer/types/colon-rectal-cancer/about/key-statistics.html Accessed 13 Jun 2024.

[2] American Cancer Society. Cancer Facts & Figures for Hispanic/Latino People 2024–2026. Atlanta: American Cancer Society, Inc.; 2024.

[3] U.S. Census Bureau. In: Quick Facts. https://www.census.gov/quickfacts/. 2022. Quick Facts (United States). Accessed 15 Jun 2024.

[4] U.S. Census Bureau. In: U.S. Census Bureau QuickFacts: Virginia. https://www.census.gov/quickfacts/fact/table/VA/PST045222. Accessed 17 Jun 2024.

[5] Virginia Department of Health. In: Cancer Incidence and mortality in Virginia. https://www.vdh.virginia.gov/data/virginia-cancer-dashboards/cancer-incidence-and-mortality-in-virginia/?utm_source=chatgpt.com. Accessed 17 Jun 2024.

[6] Gonzales M, Qeadan F, Mishra SI, Rajput A, Hoffman RM. Racial-ethnic disparities in late-stage colorectal cancer among Hispanics and non-Hispanic whites of New Mexico. Hisp Healthc Int. 2017;15(4):180–8 (**PMID: 29237342; PMCID: PMC6211799**). 10.1177/1540415317746317 PMC6211799 29237342

[7] Castañeda SF, Gallo LC, Nodora J, Talavera GA, Penedo FJ, Evenson KR, et al. Colorectal cancer screening among Hispanics/Latinos in the HCHS/SOL sociocultural ancillary study. Prev Med Rep. 2019;15(4):100947. 10.1016/j.pmedr.2019.100947. 31360630 10.1016/j.pmedr.2019.100947 PMC6639649

[8] Sanchez JI, Palacios R, Thompson B, Martinez V, O’Connell MA. Assessing colorectal cancer screening behaviors and knowledge among at-risk Hispanics in Southern New Mexico. J Cancer Ther. 2013;4(6B):15–25. 10.4236/jct.2013.46A2003. 25621179 10.4236/jct.2013.46A2003 PMC4303072

[9] Guerra ZC, Moore JR, Londoño T, Castro Y. Associations of acculturation and gender with obesity and physical activity among Latinos. Am J Health Behav. 2022;46(3):324–36. 10.5993/AJHB.46.3.11. 35794757 10.5993/AJHB.46.3.11 PMC10877675

[10] López-Suárez A. Burden of cancer attributable to obesity, type 2 diabetes and associated risk factors. Metabolism. 2019;92:136–46. 10.1016/j.metabol.2018.10.013. 30412695 10.1016/j.metabol.2018.10.013

[11] Botteri E, Iodice S, Bagnardi V, Raimondi S, Lowenfels AB, Maisonneuve P. Smoking and colorectal cancer a meta-analysis. JAMA. 2008;300(23):2765–78. 10.1001/jama.2008.839. 19088354 10.1001/jama.2008.839

[12] Clinton SK, Giovannucci EL, Hursting SD. The world cancer research fund/American Institute for cancer research third expert report on diet, nutrition, physical activity, and cancer: impact and future directions. J Nutr. 2020;150(4):663–71. 10.1093/jn/nxz268. 31758189 10.1093/jn/nxz268 PMC7317613

[13] U.S Department of Health and Human Services, Office of Disease Prevention and Health Promotion. Healthy People 2030. In: Increase the proportion of adults who get screened for colorectal cancer - C-07. https://health.gov/healthypeople/objectives-and-data/browse-objectives/cancer/increase-proportion-adults-who-get-screened-colorectal-cancer-c-07. Accessed 18 Jun 2024.

[14] Wittich AR, Shay LA, Flores B, De La Rosa EM, Mackay T, Valerio MA. Colorectal cancer screening: understanding the health literacy needs of Hispanic rural residents. AIMS Public Health. 2019;6(2):107–20. 10.3934/publichealth.2019.2.107. 31297397 10.3934/publichealth.2019.2.107 PMC6606525

[15] Jerant AF, Arellanes RE, Franks P. Factors associated with Hispanic/non-Hispanic white colorectal cancer screening disparities. J Gen Intern Med. 2008;23(8):1241–5. 10.1007/s11606-008-0666-1. 18500503 10.1007/s11606-008-0666-1 PMC2517962

[16] Diaz JA, Goldman R, Arellano N, Borkan J, Eaton CB. (2011). Brief report: exploration of colorectal cancer risk perceptions among Latinos. *Journal of immigrant and minority health*, *13*(1), 188–192. 10.1007/s10903-009-9312-117. National Cancer Institute. In: Screen to Save: NCI Colorectal Cancer Outreach and Screening Initiative. https://www.cancer.gov/about-nci/organization/crchd/community-outreach/screen-to-save. Accessed 12 May 2024. 10.1007/s10903-009-9312-1 PMC2917541 20063065

[17] National Cancer Institute. Screen to Save: NCI Colorectal Cancer Outreach and Screening Initiative [Internet]. Accessed 17 March 2024 https://www.cancer.gov/about-nci/organization/crchd/community-outreach/screen-to-save

[18] National Cancer Institute. In: National Outreach Network (NON). https://www.cancer.gov/about-nci/organization/crchd/community-outreach/non. Accessed 12 May 2024.

[19] Rafie C, Ayers A, Cadet D, Quillin J, Hackney MH. Reaching hard to reach populations with hard to communicate messages: efficacy of a breast health research champion training program. J Cancer Education: Official J Am Association Cancer Educ. 2015;30(3):599–606. 10.1007/s13187-014-0720-0. 10.1007/s13187-014-0720-0 PMC4345135 25171905

[20] Whitaker DE, Snyder FR, San Miguel-Majors SL, Bailey LO, Springfield SA. Screen to save: results from NCI’s colorectal cancer outreach and screening initiative to promote awareness and knowledge of colorectal cancer in racial/ethnic and rural populations. Cancer Epidemiology, Biomarkers & Prevention. 2020;29(5):910–7. 10.1158/1055-9965.EPI-19-0972. 10.1158/1055-9965.EPI-19-0972 PMC12306618 32358038

[21] Preston MA, Cadet D, Hunley R, Retnam R, Arezo S, Sheppard VB. Health equity and colorectal cancer awareness: a community health educator initiative. J Cancer Education: Official J Am Association Cancer Educ. 2023;38(1):225–30. 10.1007/s13187-021-02102-2. 10.1007/s13187-021-02102-2 PMC8532449 34677801

[22] Watson R, Preedy V. 5-Point Likert Scale. In: Handbook of Disease Burdens and Quality of Life Measures. V. R. Preedy, & R. R. Watson, editors, New York, NY; 2010. p. 4288.

[23] R Core Team. In: A Language and Environment for Statistical Computing. R Foundation for Statistical Computing. https://www.R-project.org/ Accessed 22 Jan 2025.

[24] Molina Y, Briant KJ, Sanchez JI, O’Connell MA, Thompson B. Knowledge and social engagement change in intention to be screened for colorectal cancer. Ethn Health. 2018;23(5):461–79. 10.1080/13557858.2017.1280135. 28116917 10.1080/13557858.2017.1280135 PMC5524622

[25] Gonzales R, Ratnapradipa K, De Alba A, Chen K, Smith L, Kim J, et al. Awareness and knowledge of colorectal cancer screening among Latinos in Omaha, Nebraska. J Immigr Minor Health. 2023;25(1):161–7. 10.1007/s10903-022-01358-0. 35357621 10.1007/s10903-022-01358-0

[26] Peterson EB, Ostroff JS, DuHamel KN, D’Agostino TA, Hernandez M, Canzona MR, et al. Impact of provider-patient communication on cancer screening adherence: a systematic review. Prev Med. 2016;93:96–105. 10.1016/j.ypmed.2016.09.034. 27687535 10.1016/j.ypmed.2016.09.034 PMC5518612

[27] Shokar NK, Salinas J, Dwivedi A. Mediators of screening uptake in a colorectal cancer screening intervention among Hispanics. BMC Cancer. 2022;22(1):37. 10.1186/s12885-021-09092-w. 34983440 10.1186/s12885-021-09092-w PMC8729110

[28] Wang J, Moehring J, Stuhr S, Krug M. Barriers to colorectal cancer screening in Hispanics in the United States: an integrative review. Appl Nurs Res. 2013;26(4):218–24. 10.1016/j.apnr.2013.08.005. 24238084 10.1016/j.apnr.2013.08.005

[29] Diaz JA, Roberts MB, Goldman RE, Weitzen S, Eaton CB. Effect of language on colorectal cancer screening among Latinos and non-Latinos. Cancer Epidemiol Biomarkers Prev. 2008;17(8):2169–73. 10.1158/1055-9965.EPI-07-2692. 18708410 10.1158/1055-9965.EPI-07-2692 PMC2568081

[30] Byrd TL, Calderón-Mora J, Salaiz R, Shokar NK. Barriers and facilitators to colorectal cancer screening within a Hispanic population. Hispan Health Care Int. 2019;17(1):23–9. 10.1177/1540415318818982. 10.1177/1540415318818982 30574791

[31] Heintzman JD, Ezekiel-Herrera DN, Quiñones AR, Lucas JA, Carroll JE, Gielbultowicz SH, et al. Disparities in colorectal cancer screening in Latinos and non-Hispanic Whites. Am J Prev Med. 2022;62(2):203–10. 10.1016/j.amepre.2021.07.009. 34649735 10.1016/j.amepre.2021.07.009 PMC11458137

[32] Castañeda SF, Xiong Y, Gallo LC, Yepes-Rios M, Ji M, Talavera AC, Mendoza PM, Talavera GA. Colorectal cancer educational intervention targeting Latino patients attending a community health center. J Prim Care Community Health. 2012;3(3):164–9. 10.1177/2150131911427731. 23803776 10.1177/2150131911427731 PMC4169886

[33] American Psychiatric Association. In: Stress & trauma toolkit for treating undocumented immigrants in a changing political and social environment. https://www.psychiatry.org/psychiatrists/diversity/education/stress-and-trauma/undocumented-immigrants?utm_source=chatgpt.com. Accessed 29 Nov 2024.

